# Genome-Wide Estimates of Runs of Homozygosity, Heterozygosity, and Genetic Load in Two Chinese Indigenous Goat Breeds

**DOI:** 10.3389/fgene.2022.774196

**Published:** 2022-04-26

**Authors:** Guixin Li, Jianhong Tang, Jinyan Huang, Yongchuang Jiang, Yin Fan, Xiaopeng Wang, Jun Ren

**Affiliations:** ^1^ Guangdong Laboratory for Lingnan Modern Agriculture, College of Animal Science, South China Agricultural University, Guangzhou, China; ^2^ Laboratory Animal Engineering Research Center of Ganzhou, Gannan Medical University, Ganzhou, China; ^3^ Department of Animal Science, Jiangxi Biotech Vocational College, Nanchang, China

**Keywords:** Chinese indigenous goats, runs of homozygosity, heterozygosity, genetic load, candidate genes

## Abstract

Runs of homozygosity (ROH) and heterozygosity (ROHet) are windows into population demographic history and adaptive evolution. Numerous studies have shown that deleterious mutations are enriched in the ROH of humans, pigs, cattle, and chickens. However, the relationship of deleterious variants to ROH and the pattern of ROHet in goats have been largely understudied. Here, 240 Guangfeng and Ganxi goats from Jiangxi Province, China, were genotyped using the Illumina GoatSNP50 BeadChip and genome-wide ROH, ROHet, and genetic load analyses were performed in the context of 32 global goat breeds. The classes with the highest percentage of ROH and ROHet were 0.5–2 Mb and 0.5–1 Mb, respectively. The results of inbreeding coefficients (based on SNP and ROH) and ROHet measurements showed that Guangfeng goats had higher genetic variability than most Chinese goats, while Ganxi goats had a high degree of inbreeding, even exceeding that of commercial goat breeds. Next, the predicted damaging homozygotes were more enriched in long ROHs, especially in Guangfeng goats. Therefore, we suggest that information on damaging alleles should also be incorporated into the design of breeding and conservation programs. A list of genes related to fecundity, growth, and environmental adaptation were identified in the ROH hotspots of two Jiangxi goats. A sense-related ROH hotspot (chromosome 12: 50.55–50.81 Mb) was shared across global goat breeds and may have undergone selection prior to goat domestication. Furthermore, an identical ROHet hotspot (chromosome 1: 132.21–132.54 Mb) containing two genes associated with embryonic development (*STAG1* and *PCCB*) was detected in domestic goat breeds worldwide. Tajima’s D and BetaScan2 statistics indicated that this region may be caused by long-term balancing selection. These findings not only provide guidance for the design of conservation strategies for Jiangxi goat breeds but also enrich our understanding of the adaptive evolution of goats.

## Introduction

Runs of homozygosity (ROH) are defined as continuous homozygous segments in the diploid genome (Gibson et al., 2006). ROH are mainly caused by demographic history, such as population bottlenecks, genetic drift, and inbreeding. Intensive natural and artificial selection has also reshaped ROH patterns in various genomic regions (Peripolli et al., 2017). ROH are considered an advanced method for quantifying and understanding inbreeding in humans and livestock (Peripolli et al., 2017; Ceballos et al., 2018). A short ROH fragment suggests ancestral inbreeding, whereas a long ROH fragment means recent inbreeding (Keller et al., 2011). The distribution of ROH fragments in chromosomes is nonrandom, and many molecular markers have unusual frequencies in ROH (Bosse et al., 2012); these are called “ROH hotspots”. An increasing number of studies have confirmed that ROH hotspots also undergo positive selection in cattle (Biscarini et al., 2020; Freitas et al., 2021), pigs (Shi et al., 2020; Gorssen et al., 2021), horses (Ablondi et al., 2020; Santos et al., 2021), sheep (Dzomba et al., 2021; Gorssen et al., 2021), and goats (Bertolini et al., 2018a; Islam et al., 2019). Positive selection tends to increase homozygosity in the target region (Xu et al., 2019) and generally promotes the spread of beneficial mutations and removes adverse ones (Makino et al., 2018). However, many deleterious mutations in linkage disequilibrium with beneficial mutations also increase in frequency due to genetic hitchhiking (Szpiech et al., 2013). Deleterious mutations are shaped by many evolutionary forces (Chun and Fay, 2011; Bortoluzzi et al., 2019), such as population decline, artificial selection, inbreeding, and genetic drift. Most mutations at functional sites are expected to be deleterious (Makino et al., 2018), and the variability and fitness of populations are largely related to the proportion of numerous slight-effect and rare large-effect deleterious alleles among the founders of these populations (Diez-Del-Molino et al., 2018). When homozygous deleterious mutations are not lethal and inbreeding is recent, mutation–selection balance will not have sufficient time to eliminate deleterious mutations in the ROH region (Szpiech et al., 2013). Therefore, previous literature revealed that deleterious mutations are more enriched in ROHs in humans (Szpiech et al., 2013), commercial pigs (Bosse et al., 2018), cattle (Zhang et al., 2015), and chicken (Bosse et al., 2018; Bortoluzzi et al., 2019). However, as far as we know, the relationship between deleterious variants and ROHs in goats has not been reported in literature yet.

Runs of heterozygosity (ROHet) have emerged recently and refer to a region of contiguous heterozygous single nucleotides detected between homologous chromosomes in diploid organisms and can provide insight into heterozygous clusters (Williams et al., 2016). These high frequencies of heterozygous regions (ROHet hotspots) demonstrate the advantages of heterozygous dominant allele expression and enhance fecundity, survival, and other fitness-related traits (Mc et al., 2009). Moreover, ROHet can be used to detect balancing selection events (Biscarini et al., 2020; Santos et al., 2021). Currently, there are few reports on ROHet in livestock. Williams et al. (2016) first introduced the concept of ROHet in livestock and suggested that some ROHet regions were under balancing selection and contained recessive lethal mutations in cattle; Santos et al. (2021) identified ROHet hotspots related to recent selection and important events in the development and performance of Mangalarga Marchador horses; while Biscarini et al. (2020) revealed that ROHet hotspots harbored the genes involved in nematode resistance and reproduction in Maremmana semi-feral cattle. However, to the best of our knowledge, ROHet have not been previously examined in goats.

Ganxi (GX) and Guangfeng (GF) goats are the only two indigenous goat breeds in Jiangxi Province of China and were originally distributed in northeastern and northwestern Jiangxi Province, respectively. The breeding history of these goats may be traced back to the Tang Dynasty (618–907 AD) (Ma et al., 2011). After long-term natural and artificial selection, these two goat breeds have formed the characteristics of small size and slow growth rate, excellent meat quality, strong disease resistance, and outstanding reproductive capability (Ma et al., 2011). GF goats have a white coat, while GX goats are mostly black (Ma et al., 2011). Although they have been recorded in the list of national conservation programs for China’s livestock and poultry genetic resources, they mainly survive in a few conservation farms and may face the risks of increased inbreeding and reduced effective population size. The assessment and quantification of the homozygotes, heterozygotes, and deleterious mutations may benefit the conservation of the Jiangxi goat breeds, but these have been largely unstudied. Therefore, the objective of this study is to characterize ROH, ROHet, and genetic load of Guangfeng and Ganxi goat breeds using the Illumina GoatSNP50 BeadChip data.

## Materials and Methods

### Ethics Statement

All animal experiments described in this study were performed in accordance with the guidelines of the Regulations for the Administration of Affairs Concerning Experimental Animals (Ministry of Science and Technology, China, revised June 2004) and approved by the Animal Care and Use Committee of South China Agricultural University, Guangzhou, China (SCAU#2013-10).

### Samples, Genotyping, and Quality Filtering

Ear tissue samples of 160 GX goats and 80 GF goats were collected from conservation farms in Jiangxi Province, China, in 2019. The individuals from each breed covered all existing consanguinities and represented the most comprehensive genetic diversity. DNA was extracted from the ear samples using the phenol/chloroform method (Sambrock and Russel, 2001). Genotyping was performed using the Illumina GoatSNP50 BeadChip (Cecchi et al., 2017), which contained 53,347 genomic SNP markers.

The reported 50K chip data of 754 animals of 31 global goat breeds, including six Chinese goat breeds (*n* = 193), seven Iranian wild goats (bezoars, BEZ), six African goat breeds (*n* = 145), nine European goat breeds (*n* = 210), three American goat breeds (*n* = 55), four Near East goat breeds (*n* = 95), and two Oceania goat breeds (*n* = 49), were downloaded (Bertolini et al., 2018b; Berihulay et al., 2019) (Supplementary Table S1). The 754 goats included 24 Guangfeng goats reported before 2019 (GF1), which were collected from different places to represent the within-breed genetic diversity (Berihulay et al., 2019). The two datasets were merged and filtered using PLINK v1.90 software (Chang et al., 2015) under the following criteria: (1) SNPs and individuals with a call rate ≥90%, (2) minor allele frequency (MAF) ≥ 0.01, and (3) all unmapped SNPs and those on sex chromosomes were eliminated. According to a previous suggestion (Martikainen et al., 2020), SNPs were not filtered based on deviations from Hardy–Weinberg equilibrium because inbreeding was one possible cause of deviation. The final dataset included 45,462 informative SNPs from 994 goats of 32 breeds for subsequent analyses.

### Identification of ROH and ROHet

ROH were identified in each goat using the consecutive method of R package *detectRUNS* (Biscarini et al., 2019) with the following parameters (Islam et al., 2019; Dadousis et al., 2021; Fabbri et al., 2021): (1) the minimum number of consecutive SNPs in an ROH was 15; (2) the minimum length of an ROH was set to 500 kb; and (3) a maximum of two SNPs with missing genotypes and up to one heterozygosity genotype were allowed in an ROH to avoid occasional genotyping errors. The sum of ROH values per goat was calculated, and five ROH estimates were classified for each goat: 0.5–2 Mb, 2–4 Mb, 4–8 Mb, 8–16 Mb, and >16 Mb (Dixit et al., 2020). The numbers for a breed in each ROH length category and proportion of ROH on each autosome were calculated.

Similarly, ROHet were estimated in consecutive stretches of heterozygous SNP genotypes with the R package *detectRUNS* (Biscarini et al., 2019). Considering that there are few reports about ROHet and no standardized criteria has been previously defined for the parameters, we used the following commands as described by previous studies (Biscarini et al., 2020; Santos et al., 2021): (1) the minimum number of consecutive SNPs included in an ROHet was 15; (2) the minimum length of an ROHet was set to 500 kb; and (3) a maximum of two SNPs with missing genotypes and up to three homozygous genotypes were allowed in an ROHet. The length of the ROHet in each individual was divided into four classes: 0.5–1* *Mb, 1–1.5 Mb, 1.5–2 Mb, and >2 Mb (Biscarini et al., 2020). The number and percentage of ROHet within each length category were calculated.

### Estimation of the Inbreeding Coefficient

Two different measures were used to calculate genomic inbreeding coefficients for each breed: (1) the SNP-based inbreeding coefficient (*F*_HOM_) was assessed using PLINK v1.90 software (Chang et al., 2015), and the formula used to calculate *F*_HOM_ was as follows: *F*_HOM_ = (*H*_O_ − *H*_E_)/(*L* − *H*_E_), where *H*_O_ is the number of observed homozygotes, *H*_*E*_ is the number of expected homozygotes, and *L* is the total number of genotyped autosomal SNPs. (2) The ROH-based inbreeding coefficient (*F*_ROH_) was estimated for each goat using the following equation (McQuillan et al., 2008): *F*_ROH_ = *L*_ROH_/*L*_auto_, where *L*_ROH_ is the total length of ROHs in the genome of each individual and *L*_auto_ is the total size of 29 autosomes of goats covered by SNPs, which was 2.46 Gb (Cardoso et al., 2018). Finally, *Pearson’s* correlation between *F*_HOM_ and *F*_ROH_ classified by each length was performed.

### Variant Annotation and Genetic Load Analyses

The Variance Effect Predictor (VEP) program in the Ensembl database (*ARS1*, http://asia.ensembl.org/Tools/VEP) was first used to identify synonymous, missense, and loss of function (LoF) mutations of 45,462 informative SNPs. LoF variants included transcript ablation, splice, stop, frame insertion, and frame deletion variants (Xue et al., 2015). Nonsynonymous SNPs with the sort intolerant from tolerance (SIFT) scores ≤0.05 were defined as deleterious mutations, otherwise regarded as tolerated mutations (Ng, 2003). Next, according to the previous description, we set bezoars (wild goat) as the outgroup population (Zheng et al., 2020). The SNP information of 24 bezoars (wild goats) was downloaded from the Goat Genome Variation, Selective Signature, and Introgression Database (http://animal.nwsuaf.edu.cn/code/index.php/GoatVar), and the ancestral state of the variant was inferred if the frequency of the allele greater than 0.5 (major homozygous) in all bezoars. Then, genetic load was computed as the ratios of deleterious to synonymous variants in each goat and averaged within each population (Bortoluzzi et al., 2019). The genetic load was calculated for homozygous derived sites, heterozygous alleles, and total deleterious alleles (heterozygotes + 2 × homozygotes).

### Enrichment of ROH for Damaging Homozygous Variants

The enrichment of damaging mutations inside and outside of ROHs was estimated using the method reported by previous studies (Szpiech et al., 2013; Bortoluzzi et al., 2019). The homozygous derived alleles were defined as damaging mutations (including deleterious and LoF variants) (Szpiech et al., 2013; Bortoluzzi et al., 2019). Previous studies classified the short ROHs with tens of kilobases (kb), medium ROHs with hundreds of kb to few megabases (Mb), and long ROHs greater than several Mb to investigate the relationship between deleterious mutations and ROHs (Pemberton et al., 2012; Szpiech et al., 2013). Considering the limitation of chip density, ROHs were divided into medium (0.5–4 Mb) and long (≥4 Mb) types in this study. Then, the occurrence of damaging mutations within each ROH length type was estimated. The individual coverage of each ROH classification was calculated by the following formula (Szpiech et al., 2013): $G_{i,j}=L_{i,j}/L_{g}$ , where $L_{i,j}$ is the total length of ROH regions of class j ∈{A,  B,  R,  N} (A, medium ROH; B, long ROH; R, all ROH; and N, out ROHs) in individual *i* and $L_{g}$ is the total length of autosomal genome. Finally, *Pearson’s* correlation analysis was performed between the fraction of mutations and genome covered by ROHs.

### Detection of ROH and ROHet Hotspots

The percentage of SNP occurrences was calculated to identify the genomic regions with a high frequency of ROH (ROH hotspots) and ROHet (ROHet hotspots). For ROHs, previous literature set thresholds for ROH hotspots at 45% (Islam et al., 2019), 26% (top 0.2%) (Bertolini et al., 2018a), and 16% (top 0.1%) (Gorssen et al., 2021) of SNP occurrence in goat populations. A previous study (Gorssen et al., 2021) suggested that the minimal threshold for ROH hotspot detection in livestock populations was set to 30% of the ROH incidences. Therefore, the genomic regions with SNP percentages exceeding the top 0.5% (33.75% in GF and 42.5% in GX) were defined as ROH hotspots in this study. In comparison, we did not find a persuasive threshold of ROHet in previous studies (Williams et al., 2016; Biscarini et al., 2020; Santos et al., 2021). In this study, the observed SNPs in ROHet at the top 0.1% level were identified as ROHet hotspots (Tsartsianidou et al., 2021). In addition, Tajima’s D values and *β* scores were calculated using VCFtools (Danecek et al., 2011) and BetaScan2 (Siewert and Voight, 2020) software, respectively, to validate candidate regions that may have undergone balancing selection. Positive values of Tajima’s D can be taken as evidence of balancing selection or population subdivision (Tajima, 1989), while *β* is expected to score above zero in the presence of long-term balancing selection (Siewert and Voight, 2020). As individual statistics may be subjected by the limitation of genotyping and missing errors in chip data and SNP distance in some goat genomes extends to approximately 200–226 kb still with some extent of linkage disequilibrium (r^2^ > 0.2) (Lashmar et al., 2015; Kumar et al., 2018; Berihulay et al., 2019); therefore, Tajima’s D and *β* statistics were estimated using a 250 kb sliding window. *P*-values were calculated for the Z-scores of Tajima’s D and *β* values using *pnorm* in R, and regions with *p* < 0.05 in the whole genome were defined as the extreme regions that may be under balancing selection.

### Candidate Gene, Pathway, and Functional Analyses

Candidate loci were annotated *via* the *Ensemble* database (*ARS1*, http://www.ensemble.org/) over 100 kb regions (50 kb upstream and 50 kb downstream). Gene Ontology (GO) terms and Kyoto Encyclopedia of Genes and Genomes (KEGG) pathways were explored for functional enrichments of the candidate genes using the *Metascape* website (https://metascape.org/).

## Results

### Genomic Distribution of ROH and ROHet

**FIGURE 1:**
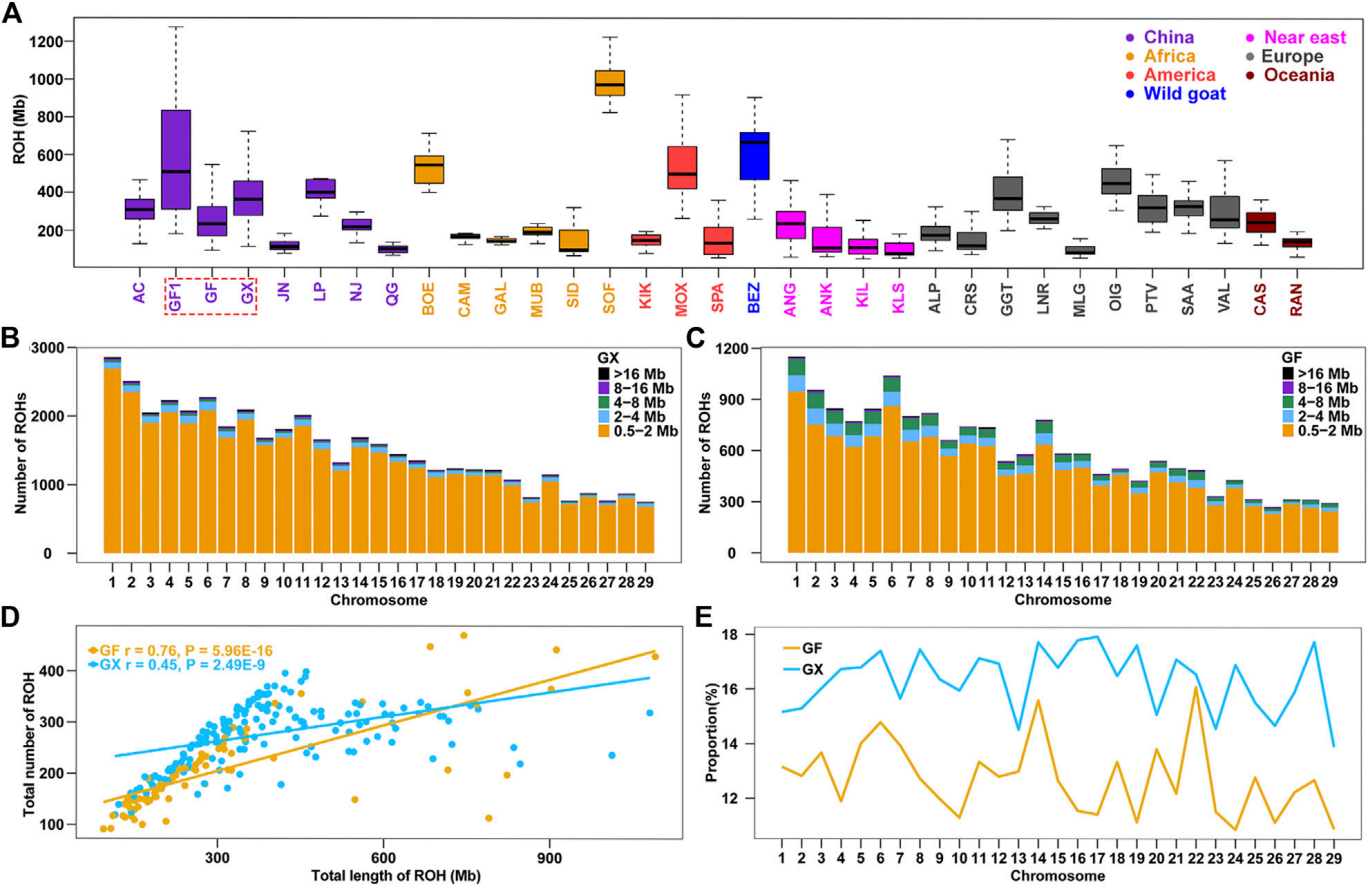
Summary results of ROH. **(A)** Boxplot of total ROH (Mb) length of individuals from 32 goat populations. Three Jiangxi native populations, GF1, GF, and GX, are indicated by colored wireframe. **(B–C)** Number distribution of five different lengths of ROH on each autosome in GX and GF goats. **(D)** Total number of ROHs plotted with the total length of ROHs per animal in GX and GF goats. **(E)** Average percentage of each autosome covered by ROH in GF and GX goats. Abbreviations of all breeds are given in Supplementary Table S1.

Genome-wide ROHs were estimated on 29 autosomes in 32 global goat breeds. The average ROH length ranged from 120.10 Mb (Chinese Qingeda goats, QG) to 996.40 Mb (African Sofia goats, SOF), and Chinese autochthonic goat breeds displayed higher levels of ROHs than other worldwide goat breeds (*p* = 0.01, ANOVA) (Supplementary Table S1; Supplementary Table S2). The ROH length in GF goats (309.4 Mb) was less than that in early-day Guangfeng goats (GF1, ROH = 579.10 Mb) (Figure 1A), indicating that the genetic variability in current GF goats is significantly boosted (*p* < 0.001, ANOVA). GX goats had a larger ROH length (393.20 Mb) in Chinese goats, even longer than that of the modern Angora (ANG, ROH = 251.80 Mb) and Saanen dairy (SAA, ROH = 315.70 Mb) goats, which have undergone intensive selection in wool and milk production (Figure 1A). A total of 44,422 and 16,598 ROH segments were detected in the GX and GF breeds, with averages of 277.60 and 207.50 ROH segments detected for each GX and GF goat, respectively. The number of short fragments of ROH (0.5–2 Mb) accounted for the largest percentage of both total ROH segments and all autosomes (Figure 1B; Figure 1C). A significant correlation between the number and total length of ROH in each individual was estimated in GX (r = 0.76, *p* = 5.96 × 10^−16^) and GF (r = 0.45, *p* = 2.49 × 10^−9^) goats (Figure 1D). In the two current Jiangxi goat breeds, although the average ROH length in GF goats was less than that in GX goats, GF goats had an individual with the largest ROH length compared to GX goats (Figure 1D; Supplementary Table S2). The variation coefficient (CV) of ROH length in GF goats (0.70) was greater than that in GX (0.43) and GF1 (0.55) goats (Supplementary Table S1), revealing the large difference of inbreeding degree in GF goats. The ratio of the ROH length on each autosome was calculated, and the results indicated that the ratios of GX goats were always greater than those of GF goats on 29 autosomes, and the differences in the two breeds ranged from 0.47% (chromosome (CHR) 22) to 6.51% (CHR17) (Figure 1E).

**FIGURE 2:**
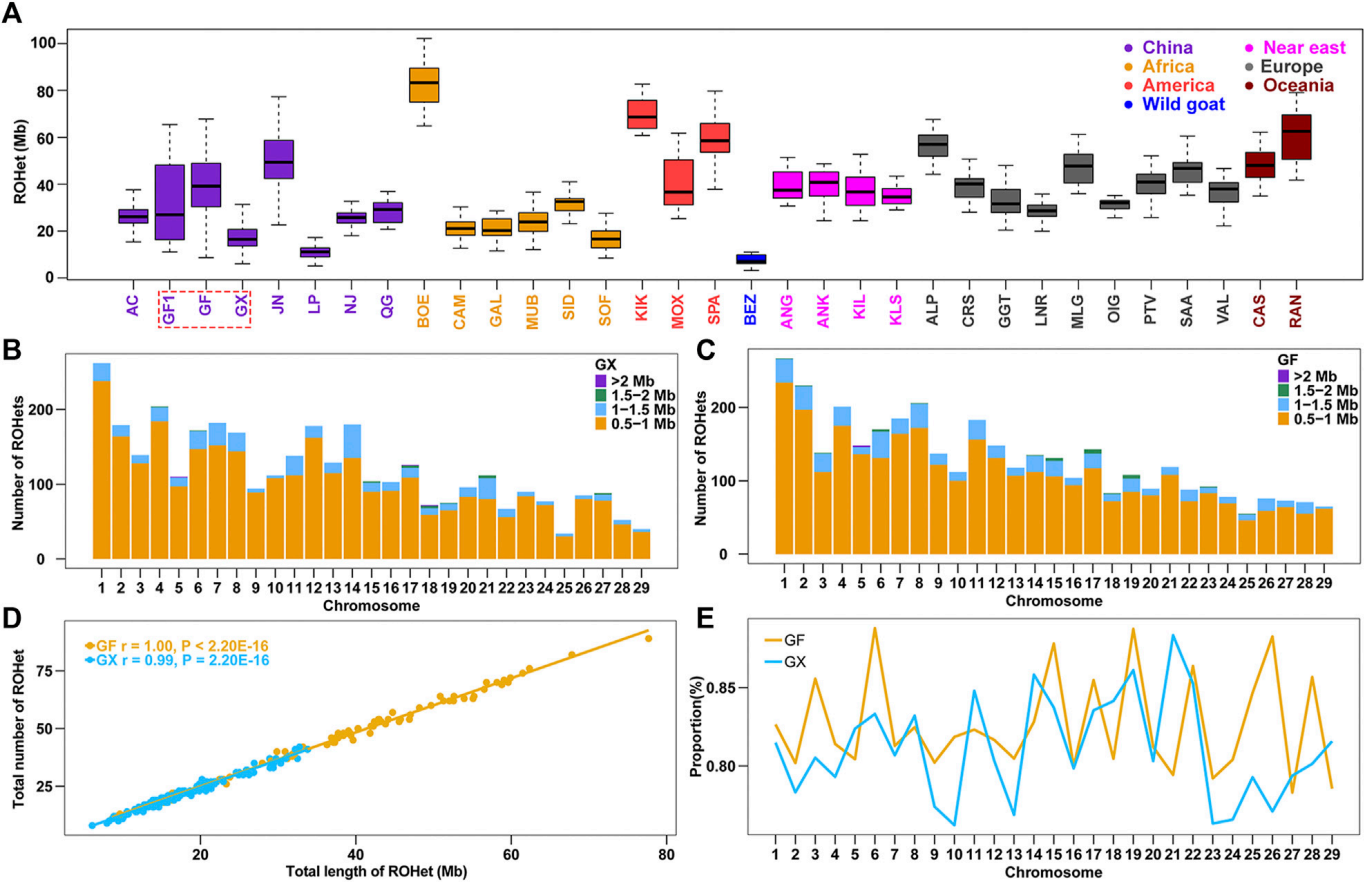
Summary results of ROHet. **(A)** Boxplot of total ROHet (Mb) length of individuals from 32 goat populations. Three Jiangxi native populations, GF1, GF, and GX, are indicated by colored wireframe. **(B–C)** Number distribution of four different lengths of ROHet in each autosome of GX and GF goats. **(D)** Total number of ROHet plotted with the total length of ROHet per animal in GX and GF goats. **(E)** Average percentage of each autosome covered by ROHet in GF and GX goats. Abbreviations of all breeds are given in Supplementary Table S1.

Similarly, the ROHet were also counted among the 32 tested goat breeds. The average length of ROHet ranged from Chinese Louping goats (LP, ROHet = 11.30 Mb) to commercial Boer goats (BOE, ROHet = 82.80 Mb) among the 31 domesticated goat breeds (Supplementary Table S1), and the average ROHet length in Chinese goat breeds were normally less than that of other worldwide goats (*p* < 0.05, ANOVA; Figure 2A; Supplementary Table S1). It is interesting to note that we identified fewer ROHet in 160 GX goats (*n* = 3,469) than in 80 GF goats (*n* = 3,753). GX goats had the second lowest ROHet length (17.6 Mb) in Chinese goats, which may be related to their high level of inbreeding (the third largest ROH length in Chinese goats) (Supplementary Table S1). In contrast, GF goats harbored higher ROHet levels (38.8 Mb) than GX (*p* = 1.96 × 10^−21^, ANOVA) and GF1 (30.4 Mb, *p* = 0.01, ANOVA) goats, which was consistent with the decreased ROH level in GF goats. Similar to ROH, the number of short ROHet fragments (0.5–1 Mb) was higher than other categories, and the amount of ROHet in each autosome increased with chromosome length (*p* < 0.001, ANOVA) in GX and GF goat breeds (Figure 2B; Figure 2C). The number of ROHet showed a significant positive correlation with the total length of ROHet per individual in GX (r = 0.99, *p* < 2.20 × 10^−16^) and GF (r = 1.00, *p* < 2.20 × 10^−16^) goats (Figure 2D). The majority of GF individuals harbored a higher total number and length of ROHet than GX individuals, indicating abundant genomic heterozygosity in GF goats (Figure 2D; Supplementary Table S2). GF1 (CV_ROHet_ = 0.55) and GF (CV_ROHet_ = 0.38) goats had the largest and third largest coefficients of variation in global goat breeds (Supplementary Table S1), respectively, also revealing the complex demographic histories of Guangfeng goats. The highest genome coverage of ROHet was detected on CHR6 in GF goats (0.89%), while the lowest ROHet coverage was located on CHR10 in GX goats (0.76%) (Figure 2E). Different from the abovementioned ROH results, the ROHet ratios of GX goats contained eight autosomes (CHR5, 8, 11, 14, 18, 21, 27, and 29), higher than that of GF goats (Figure 2E).

### Inbreeding Coefficients of Guangfeng and Ganxi Goats

**FIGURE 3:**
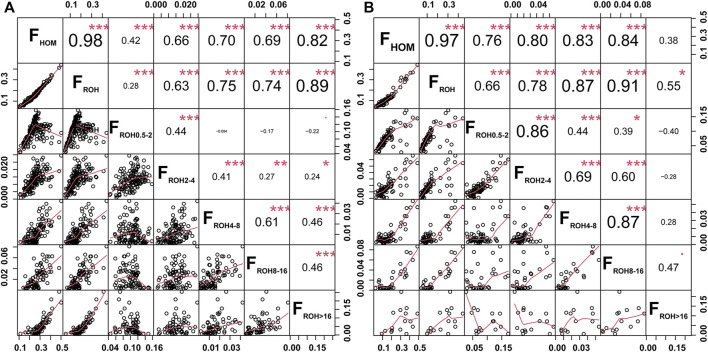
Scatterplots (lower triangle) and correlations (upper triangle) of the genomic inbreeding coefficients between the *F*_HOM_ and *F*_ROH_ (*F*_ROH_; *F*_ROH 0.5–2 Mb_; *F*_ROH 2–4 Mb_; *F*_ROH 4–8 Mb_; *F*_ROH 8–16 Mb_; *F*_ROH >16 Mb_) in GX **(A)** and GF **(B)** goats. *P < 0.05, **p < 0.01, and ***p < 0.001.

Two inbreeding coefficients were calculated based on the genomic data of all tested individuals. The *F*_HOM_ showed a significant positive correlation with the average number of ROHs (r = 0.91, *p* = 1.48 × 10^−13^) and *F*_ROH_ (r = 0.89, *p* = 8.10 × 10^−12^) in global goat breeds. The average *F*_HOM_ and *F*_ROH_ in Chinese goats (*F*_HOM_ = 0.18_,_ *F*_ROH_ = 0.13) were higher than those in other goat breeds worldwide (*F*_HOM_ = 0.14_,_ *F*_ROH_ = 0.12) (Supplementary Table S1). For Jiangxi goats, GF1 had the highest *F*_ROH_ (0.24) and second highest *F*_HOM_ (0.27) values among Chinese local goat breeds, and the *F*_HOM_ was only lower than SOF (*F*_HOM_ = 0.44) in domesticated goats worldwide. In contrast, GF goats had lower values of *F*_HOM_ (0.16) and *F*_ROH_ (0.13) than GF1 goats and presented a moderate degree of inbreeding among the 32 goat breeds. GX goats exhibited a higher level of inbreeding degree, reflected in the fifth and ninth greatest values of *F*_HOM_ and *F*_ROH_, than that of other global goat breeds (Supplementary Table S1). Significant positive correlations were also detected between the *F*_HOM_ and *F*_ROH_ in GX (r = 0.98, *p* < 0.001) and GF (r = 0.97, *p* < 0.001) goats (Figure 3). The *F*_ROH_ values were subdivided into five classes, and *F*_ROH>16_ and *F*_ROH8-16_ displayed the highest significant correlation coefficients with the *F*_ROH_ in GX (r = 0.89, *p* < 0.001) and GF (r = 0.91, *p* < 0.001) goats (Figure 3), respectively. According to the formula *L*_ROH_ = 100/(2g*cM), *g* is the generation in the past, and 1 cM is approximately equal to 1 Mb (Peripolli et al., 2018). This inferred that inbreeding events in GX and GF goats occurred mainly within the last three and three to six generations.

### Genetic Load for Deleterious Mutations

**FIGURE 4:**
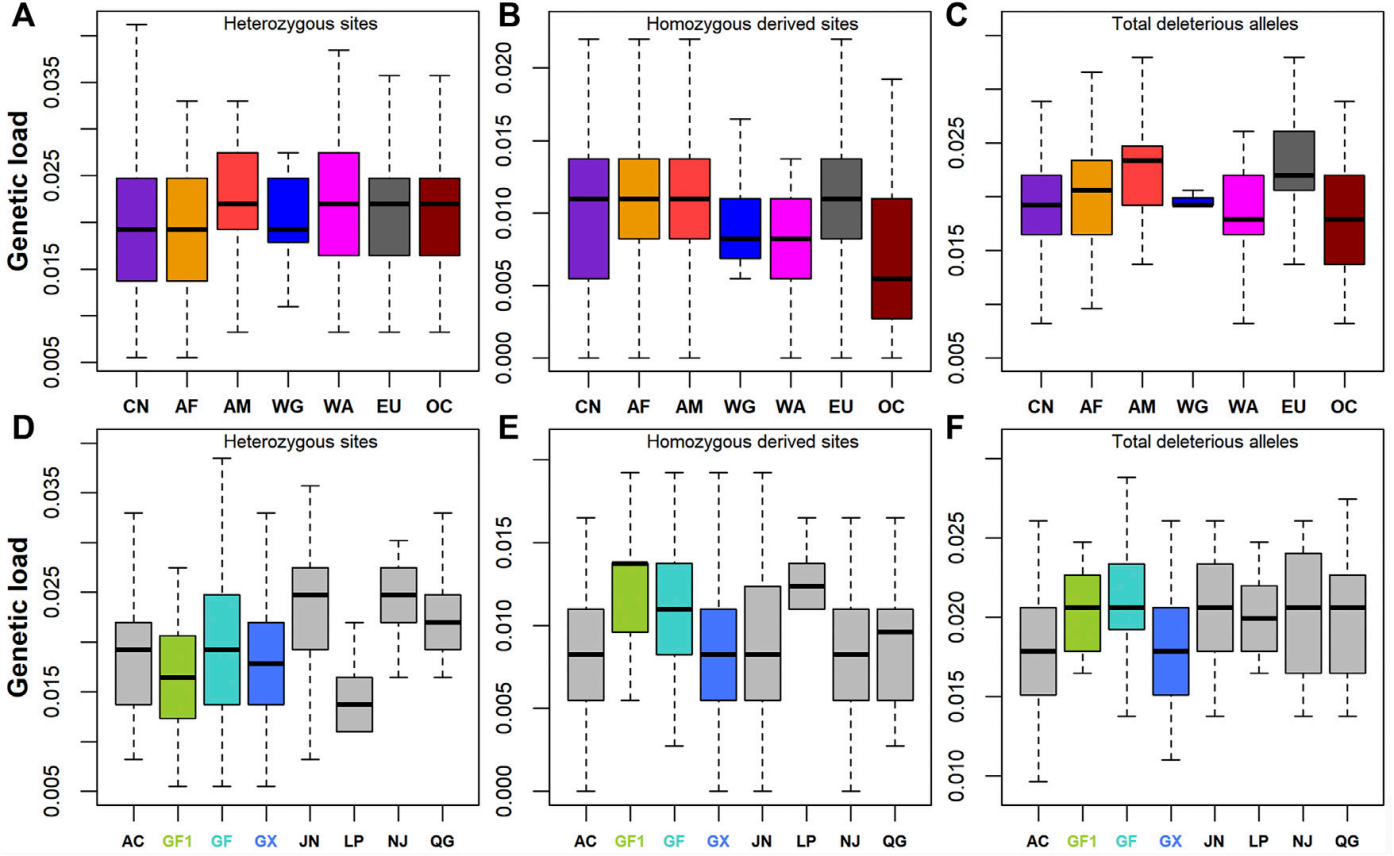
Genetic load defined as deleterious to synonymous ratio. **(A–C)** Genetic load for heterozygotes, deleterious homozygotes, and total deleterious alleles across seven global groups, respectively. **(D–F)** Genetic load for heterozygotes, deleterious homozygotes, and total deleterious alleles across eight Chinese goat populations, respectively. Abbreviations of all breeds and groups are given in Supplementary Table S1.

A total of 364, 159, and 46 synonymous, missense, and LoF variants were identified, respectively. Nonsynonymous variants included 21 deleterious variants with SIFT ≤0.05. Genetic load was then calculated based on the ratios of heterozygotes, homozygous-derived sites, and total deleterious alleles to synonymous sites/alleles. The results showed that Chinese goats had low levels of genetic load for heterozygous sites (Figure 4A) and total deleterious alleles (Figure 4C; Supplementary Table S1). On the contrary, the genetic load of homozygous-derived sites was, on average, higher in Chinese goats among global goat breeds. The large differences among Chinese goat breeds were manifested by the higher fluctuation of genetic load for homozygous derived sites (Figure 4B). Among Chinese goats, southern Chinese goat breeds (including GF1, GF, GX, and LP) had lower levels of genetic load for heterozygous sites (Figure 4D) and higher levels of genetic load for homozygous-derived sites (Figure 4E) than that of northern Chinese goat breeds (including AC, JN, NJ, and QG) (Supplementary Table S1). This outcome was in line with the pattern of the ROH and ROHet. In Jiangxi goat breeds, the genetic load values of total deleterious alleles (Figure 4F) and homozygous-derived sites were greater in GF1 and GF than GX goats. In addition, although GF goats harbored a lower degree of inbreeding than GX goats, all the three types of genetic load were larger than that in GX goats.

### Damaging Mutations More Enriched in Long ROH

**FIGURE 5:**
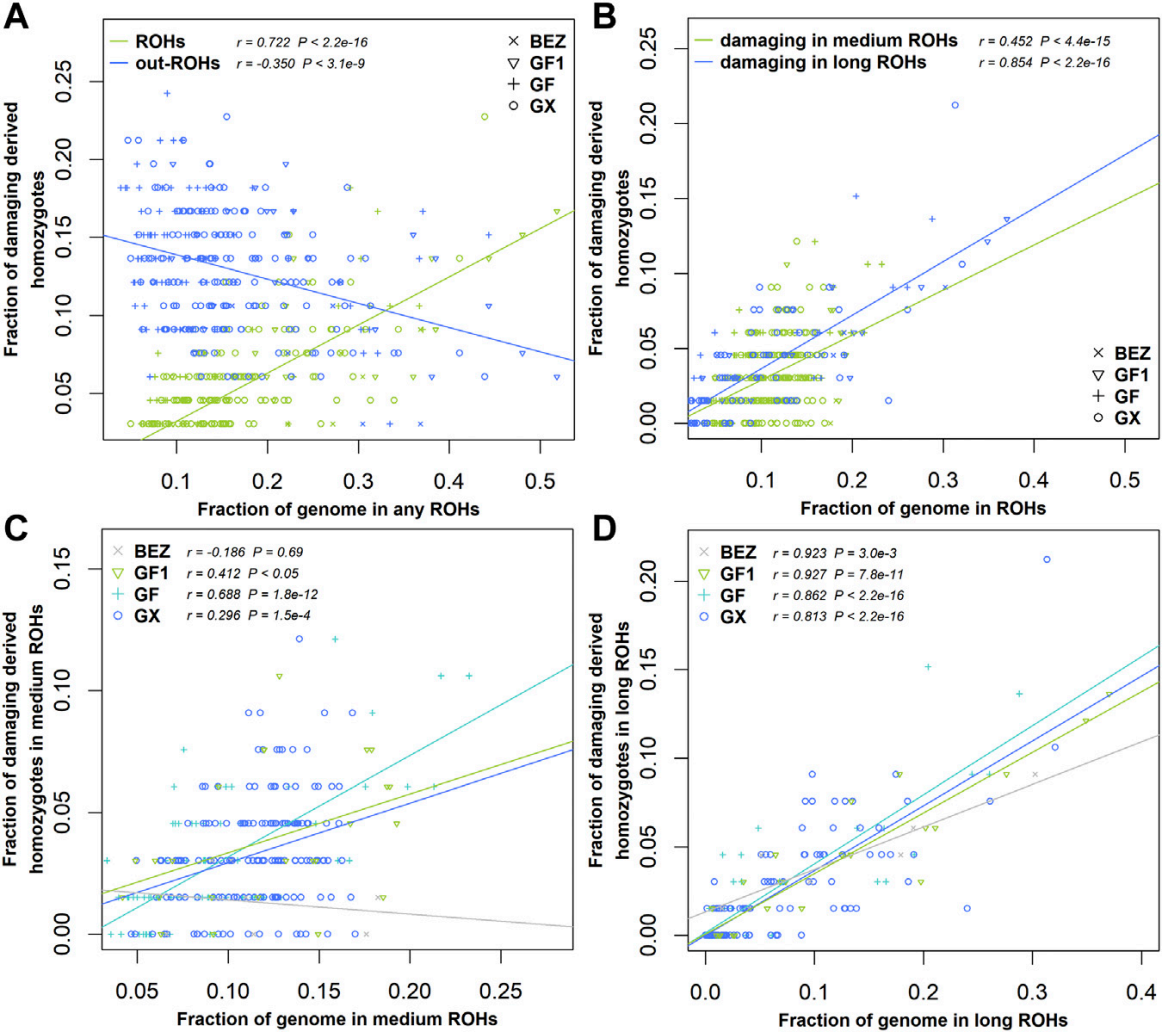
Relationship between damaging mutations and ROH. **(A)** Fraction of all genome-wide damaging derived homozygotes falling inside and outside the ROH regions versus the genome covered by ROHs. **(B)** Fraction of damaging derived homozygotes versus the fraction of the genome covered by medium and long ROHs for all individuals of four populations. **(C)** Fraction of damaging derived homozygotes versus the fraction of the genome covered by medium or **(D)** long ROHs in each population. Abbreviations of all breeds are given in Supplementary Table S1.

ROHs are widely used to investigate demographic history. In this study, we estimated the enrichment of damaging variants in different ROH length classifications of BEZ, GF1, GF, and GX goats. The fraction of homozygous-derived sites for damaging variants within each ROH length type enhanced with the increasing of genome coverage in each ROH length classification (Figure 5). A negative correlation (r = -0.35, *p* = 3.10 × 10^−9^) was observed between homozygous-derived variants and genome covered by the outside of ROH regions for damaging mutations (Figure 5A). The fraction of predicted damaging homozygotes was higher in long ROHs (r = 0.85, *p* < 2.20 × 10^−16^) than that in medium ROHs (r = 0.45, *p* < 2.20 × 10^−16^) (Figure 5B), indicating that damaging derived homozygotes are more enriched in long haplotypes. This pattern also occurred in BEZ, GF1, GF, and GX goats (Supplementary Figure S1). As expected, the levels of genetic load based on damaging homozygotes in three Jiangxi populations were higher than those in BEZ in both medium (Figure 5C) and long (Figure 5D) ROHs. Among these goat breeds, the proportion of damaging homozygotes in GF goats was higher than that in other populations, reflecting the accumulation of more harmful mutations in GF goats.

### Candidate Genes and Biological Processes in ROH Hotspots of Guangfeng and Ganxi Goats

**FIGURE 6:**
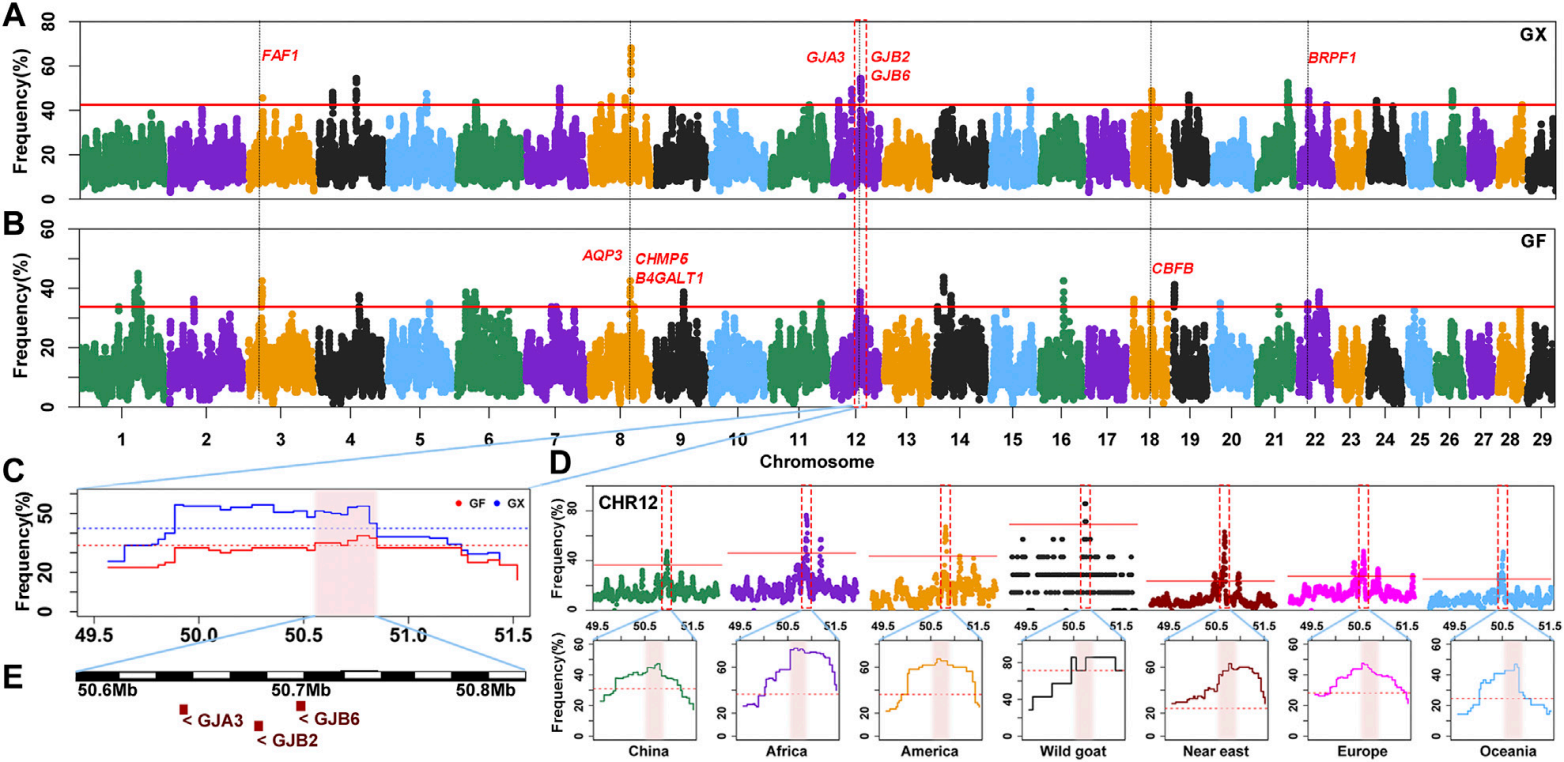
Frequency distribution of ROH in genomic regions. **(A–B)** Manhattan plot of the occurrence of each SNP in ROH in GX and GF goats. The red ablines correspond to the significance level threshold (top 0.5%). Nine overlapping candidate genes are indicated in red font. **(C)** ROH hotspot shared in GX and GF goats on CHR12 (50.55–50.81 Mb) is identified with the pink background. The blue and red ablines indicate the threshold (top 0.5%) in GX and GF goats, respectively. **(D)** ROH hotspot shared in seven global goat groups on CHR12 (50.55–50.81 Mb) marked on the pink background. The red lines represent the top 0.5% threshold. **(E)** Candidate genes in the overlapping ROH hotspot.

The occurrence frequency of SNPs in the identified ROH was counted in 160 GX goats and 80 GF goats. The regions with the top 0.5% of the most frequently occurring SNPs (ROH hotspots) were defined as candidate regions under selection. The threshold of SNP occurrence was 42.5% in GX goats, and 23 separate ROH hotspots were detected, including 187 SNPs on sixteen autosomes as candidate loci (Figure 6A; Supplementary Figure S2). A total of 181 candidate genes were located in 100-kb regions surrounding the 187 SNPs. GO and KEGG analyses indicated that many genes were significantly (*p* < 0.05) enriched in the following terms: organization development (GO:0007420, GO:0043588, and GO:0007498), metabolism (GO:0004771 and GO:0051121), immunity response (GO:0006935 and ko05100) and odontogenesis (GO:0042476) (Supplementary Table S3). Five candidate genes were associated with growth development (*FAF1*, *TIMP3*, *KDM3B*, *CHMP5*, and *CBFB*), eight candidate genes were relevant to reproduction (*FAF1*, *WNT2*, *EGR1*, *B4GALT1*, *CHMP5*, *KDM6B*, *PER1*, and *BRPF1*), two genes were related to fat deposition (*TK2* and *CIDEC*), and three genes were involved in immunoreactions (*AQP3*, *FBXO7*, and *ACOD1*) (Table 1; Supplementary Table S4).

**TABLE 1:** Candidate genes of shared ROH and ROHet hotspots in Ganxi and Guangfeng goats.

Position (Mb)	Gene	Phenotype	Breed	Reference
ROH hotspots				
3:24.99–25.47	*FAF1*	Skeletal development and embryonic viability	a and b	Rangkasenee et al. (2013), Peng et al. (2017)
8:74.66–74.71	*B4GALT1*	Reproduction	a and b	Lu et al. (1997)
8:74.83–74.84	*CHMP5*	Skeletal development and reproduction	a and b	Greenblatt et al. (2015), Shim et al. (2006)
8:74.98–74.99	*AQP3*	Immunity	a and b	Thiagarajah et al. (2017)
12:50.64–50.64	*GJA3*	Vision	a, b, and c	Berthoud et al. (2014)
12:50.68–50.68	*GJB2*	Hearing	a, b, and c	Prera et al. (2014)
12:50.69–50.70	*GJB6*	Hearing	a, b, and c	Teubner, (2003)
18:35.97–36.02	*CBFB*	Skeletal development	a and b	Miller et al. (2002)
22:16.79–16.8	*BRPF1*	Embryonic viability	a and b	You et al. (2015)
ROHet hotspots				
1:132.15–132.48	*STAG1*	Reproduction	a, b, and c	Remeseiro et al. (2012)
1:132.45–132.59	*PCCB*	Embryonic development	a, b, and c	Ortega et al. (2017)
14:16.89–16.89	*OSR2*	Skeletal development and tooth development	a and b	Fu et al. (2017), Zhou et al. (2011)

a, Ganxi goats; b, Guangfeng goats; c, global goat breeds.

In GF goats, the threshold level of the occurrence time was 33.75%, and 29 ROH hotspots containing 281 SNPs on eighteen autosomes as candidate loci were acquired (Figure 6B; Supplementary Figure S2). A total of 125 candidate genes were annotated within 100-kb regions surrounding the 281 SNPs. Several biological processes related to metabolism (GO:0004771 and GO:0051603), ear development (GO:0048839), visual perception (GO:0007601), and osteoblast differentiation (GO:0001649, GO:0048736, and GO:0060173) (Supplementary Table S3) were detected. These candidate genes contained three growth-related genes (*FAF1*, *CHMP5*, and *CBFB*), six fecundity-related genes (*ICA1L*, *FAF1*, *B4GALT1*, *CHMP5*, *DENND1A*, and *BRPF1*), two immunity-related genes (*EPS15* and *AQP3*), two genes related to fat deposition (*PPP3CA* and *ARHGAP5*), five genes involved in ear development (*HES1*, *RELN*, *SLC26A5*, *GJB2*, and *GJB6*) and four genes associated with visual perception (*LRIT3*, *GJA3*, *CNGB3*, and *ATXN7*) (Table 1; Supplementary Table S4).

Moreover, 42 overlapping SNPs residing in six ROH hotspots on six autosomes (CHR3, 6, 8, 12, 18, and 22) between GX and GF goats were identified (Figure 6; Supplementary Figure S2). The identical regions covered 1.97 Mb of the genome and ranged from 0.11 to 0.46 Mb. A total of 52 overlapping genes were retrieved, and most genes were significantly (*p* < 0.05) enriched in GO terms associated with metabolism (GO:0004771, GO:0043966, GO:0006790, and GO:0006631) and stress response (GO:1990349, GO:0006954, GO:0097530, GO:0071396, and GO:0006979) (Supplementary Table S3). Intriguingly, one ROH hotspot on CHR12 (50.55–50.81 Mb) was shared by global goat breeds (including wild goats), and genes related to vision (*GJA3*) and hearing (*GJB2*, *GJB6*) located in this region may be crucial for adaptive evolution in goats (Figure 6D; Figure 6E).

### Candidate Genes in ROHet Hotspots of Guangfeng and Ganxi Goats

**FIGURE 7:**
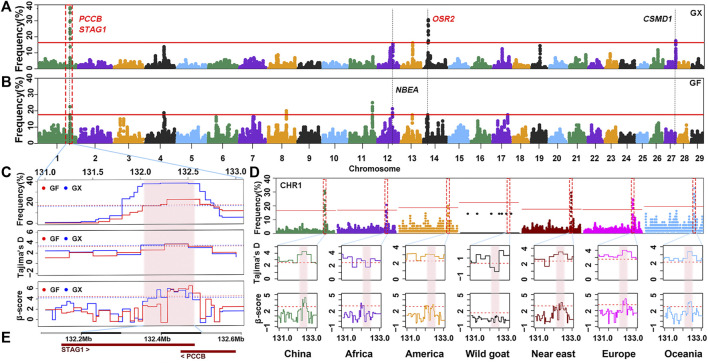
Frequency distribution of ROHet in genomic regions. **(A–B)** Manhattan plot of the incidence of each SNP in ROHet in GX and GF goats. The red ablines correspond to the significance level threshold (top 0.1%). Five overlapping and specific candidate genes are indicated by red and black fonts, respectively. **(C)** Identical region (1:132.21–132.54 Mb) was significant in the ROHet, Tajima’s D, and BetaScan2 methods in GX and GF goats. The significant region is colored by a pink background. Red and blue ablines indicate the thresholds of ROHet (top 0.1%), Tajima’s D, and BetaScan2 (statistic values corresponding to *p* < 0.05) in GX and GF goats, respectively. **(D)** Identical region (1:132.21–132.54 Mb) was significant in the ROHet, Tajima’s D, and BetaScan2 statistics in global domesticated goat breeds, except for wild goats. Red ablines represent the threshold of ROHet (top 0.1%), Tajima’s *D*, and BetaScan2 (statistic values corresponding to *p* < 0.05) in global goat groups. **(E)** Candidate genes in the shared ROHet hotspot.

Although the proportion of SNPs within an ROHet region did not surpass 50% on any chromosome, uncommonly occurred heterozygous clusters can still be found within some autosomes. ROHet hotspots were identified by including the top 0.1% of SNPs with occurrence frequencies that correspond to frequency thresholds of 17.50% and 16.25% in GX and GF goats (Figure 7A; Figure 7B), respectively. Twelve candidate genes were revealed in three heterozygous regions on CHR1, CHR14, and CHR27 in GX goats. Of note, three genes (*STAG1*, *PCCB*, and *CSMD1*) were associated with fertility, and one gene (*OSR2*) was associated with skeletal and tooth development (Table 1; Supplementary Table S4). In GF goats, 22 candidate genes on three autosomes were explored, including two reproduction-related genes (*STAG1* and *PCCB*) and two growth-related genes (*OSR2* and *NBEA*). Two common ROHet hotspots were observed on the 0.47 Mb region of CHR1 and one SNP on CHR14 (Supplementary Figure S2). Strikingly, a universal high-frequency region of an ROHet was detected on CHR1 (132.21–132.54 Mb) among the global domesticated goat breeds (Figure 7D). Furthermore, Tajima’s D and *β* scores for this region exceeded or neared the threshold lines (statistic values corresponding to *p* < 0.05) in goat breeds worldwide, except for bezoars (Figures 7C,D; Supplementary Table S5). Two candidate genes (*STAG1* and *PCCB*) residing in this region (Figure 7E) experienced balancing selection in domesticated goat breeds and likely played significant roles in goat domestication.

## Discussion

### Runs of Homozygosity and Heterozygosity Detection

The existence of ROH and ROHet could partially illustrate the results of stochastic processes across the genome. Currently, an increasing number of studies have demonstrated that the existence of such regions not only sheds light on demographic history but can also be implemented to investigate genetic diversity and adaptive evolution (Ceballos et al., 2018). Considering the estimated statistics using a window or sliding window can cause some analytical bias; hence, the consecutive run test was applied, which has been suggested to be more accurate (Santos et al., 2021) to detect genome-wide ROH and ROHet. Due to diverse computational parameters, tested populations, SNP arrays, and the number and length of ROH and ROHet obtained herein were different than those obtained from previous studies in cattle (Biscarini et al., 2020), turkeys (Marras et al., 2018), horses (Santos et al., 2021), and sheep (Tsartsianidou et al., 2021). For example, the average number of ROHet detected per animal (45.50 in this study) was higher than that detected by other studies in cattle (9.87) (Biscarini et al., 2020) and sheep (28.28) (Tsartsianidou et al., 2021) and lower than that detected in turkeys (57.80) (Marras et al., 2018) and horses (52.17) (Santos et al., 2021). Nevertheless, our results were consistent with these studies wherein the short segment of ROH (<2 Mb) and ROHet (<1 Mb) showed the largest proportion, and ROHet were much rarer and shorter than ROH. In general, the average number and length of ROH were greater in Chinese goat breeds than in other tested goat breeds, while the opposite was true for ROHet. Focusing on Jiangxi goat breeds, moderate *Pearson’s* correlation coefficients between the length of ROH and ROHet were present in GX (r = −0.61, *p* < 2.20 × 10^−16^) and GF (r = −0.56, *p* < 2.20 × 10^−16^) goats. In addition, despite the average count and length of ROHet in GF goats being higher than those in GX goats, the coverage rates of the ROHet in GF goats were not always greater than those in GX goats on 29 autosomes, which is dissimilar to the result of ROH. Considering that the number of heterozygotes is lower than that of homozygotes in the species genome, the detection of ROHet may be more susceptible to SNP density than that of ROH. As expected, the average number (*p* < 0.01, ANOVA) and length (*p* = 1.50 × 10^−11^, ANOVA) of ROH were significantly greater than those of ROHet in the 32 tested populations. The incidence of SNPs in ROH was significantly higher than that in ROHet (*p* = 7.80 × 10^−46^ in GF goats, ANOVA) in both Jiangxi goat breeds, especially GX goats (*p* = 7.75 × 10^−58^, ANOVA) (Supplementary Figure S3). It was expected that the occurrence of ROH should have a significant negative correlation with ROHet. However, the results showed that they were not negatively correlated but showed an extremely weak and significant positive correlation in GF (r = 0.074, *p* < 2.20 × 10^−16^) and GX (r = 0.049, *p* < 2.20 × 10^−16^) goats. This discrepancy may be due to the limitation of SNP density and the few missing and homozygous genotypes allowed in ROHet segments. It is worth mentioning that the detection of ROHet and the selected parameters have not been extensively studied yet (Tsartsianidou et al., 2021). Therefore, further analyses are required to improve the parameter settings of ROHet detection using high-density SNPs, more parameters, and populations. The large variation coefficients of ROH and ROHet provide options for inbreeding management in GX and GF goats, such as reducing mating opportunities between individuals with high levels of ROH or low levels of ROHet.

### Inbreeding Level in Two Jiangxi Goat Breeds

The inbreeding coefficient is a considerable parameter for monitoring population genetic variability and livestock management. In this study, the inbreeding coefficients estimated using SNPs (*F*_HOM_) were larger than those estimated using ROH (*F*_ROH_). This was consistent with the results reported in other animals, such as pigs (Shi et al., 2020) and sheep (Freitas et al., 2021), and possibly caused by the inability of the *F*_HOM_ to differentiate the identical by descent (IBD) alleles from identical by state (IBS) alleles (Wang, 2014). Our results uncovered that Chinese goat breeds had higher levels of inbreeding than other global goat breeds, including some international commercial breeds (Boer, Angora, and Saanen goats) that are undergoing heavy selection for economic traits. This indicated that Chinese goat breeds had lower genetic variation than global goat breeds. However, we cannot rule out ascertainment bias caused by sample size and the Illumina GoatSNP50 BeadChip (Tosser-Klopp et al., 2016) designed using a non-Chinese goat background. The SNP panel used in this study was developed based on the sequence data of diverse goat breeds, including Alpine, Boer, Creole, Katjang, Saanen, and Savanna goats (Tosser-Klopp et al., 2016), which reduced the SNP ascertainment bias to a certain extent. Focusing on Jiangxi goat breeds, the current Guangfeng population had lower values of *F*_ROH_ and *F*_HOM_ than the Ganxi population and previous Guangfeng population. The increased genomic heterozygosity possibly resulted from two reasons: (a) all the core individuals from the current Guangfeng population were sampled, and this population contains the most comprehensive genetic diversity; and (b) it was reported that Guangfeng goats were admixed with commercial goats (Fan et al., 2010), and the population may not have been well-managed, consequently increasing admixture and genomic variability. In contrast, Ganxi goats had a higher inbreeding degree than other Chinese goat breeds. The largest *Pearson’s* correlation coefficient was observed between the *F*_ROH_ and *F*_ROH8-16_ and *F*_ROH>16_ in GX and GF goats, respectively. Considering 4 years as one generation, more inbreeding events occurred within the last 12 and 12–24 years in GX and GF goats, respectively. It was reported that there were no conservation farms for Ganxi and Guangfeng goats before 2006 (Ma et al., 2011). Over the next 15 years, although GX and GF goats were conserved and bred on conservation farms, the inbreeding degree of the two breeds increased, probably due to closed breeding in small populations and imperfect management. Autozygosity is often involved in inbreeding depression. To protect these two precious goat germplasm resources in Jiangxi Province, China, more scientific conservation strategies and efficient management systems should be established in the future.

### Genetic Load Information May Benefit the Control of Inbreeding Depression in Jiangxi Goats

Investigating deleterious variation in the genome provides new insight into conservation genetics (Hohenlohe et al., 2021). The frequencies of deleterious alleles can be increased and fixed by genetic drift, inbreeding and demographic bottleneck, and reducing individual fitness (Bortoluzzi et al., 2019; Hohenlohe et al., 2021). In this work, Chinese goats exhibited a slightly higher genetic load of homozygous-derived variants than other global goat breeds, likely related to the larger levels of inbreeding mentioned previously. GF goats had large levels of genetic load, which may be ascribed to the demographic bottleneck in the middle of last century (Ma et al., 2011). The recent population contraction can severely affect the genetic diversity and genetic load (Bortoluzzi et al., 2019), and the genetic effect of this bottleneck can linger after demographic recovery (Hohenlohe et al., 2021). As a result, GF1 and GF goats had a high degree of inbreeding and genetic load for homozygous-derived alleles. However, GF goats showed more damaging heterozygotes and genetic diversity than those of GX goats, probably caused by recent gene flow (Fan et al., 2010) because gene flow may convert parts of homozygotes (including deleterious homozygotes) into heterozygotes in the recipient population. In agreement with previous studies in humans (Szpiech et al., 2013), pigs (Bosse et al., 2018), and chicken (Bosse et al., 2018; Bortoluzzi et al., 2019), damaging homozygotes are more enriched in ROHs than the rest of the goat genome. ROH lengths reflect the demographic history, short ROHs represent ancient relatedness, and long ROHs denote recent parental relatedness (Szpiech et al., 2013). The fraction of damaging homozygotes in long ROHs is higher than that of the corresponding medium ROHs in Jiangxi goats, indicating that the harmful mutations have higher generation in recent demographic contractions. Previous literature suggested that effective breeding programs counterbalance the negative effects of inbreeding and intensive artificial selection (Derks et al., 2018; Bortoluzzi et al., 2019). However, in native goat breeds, there is lack of special conservation, breeding, and artificial selection schemes to reduce the accumulation of genetic load. Considering that the deleterious burden was positively related to the length of ROHs, genomic deleterious variants should be purged by avoiding inbreeding in future breeding and conservation programs (Cara et al., 2013). It should be noted that although our results were in line with those of previous studies, the limitation of SNP density may have a certain impact on the precision of the outcomes. Further in-depth investigation using re-sequencing data is needed to verify and improve our results.

### ROH Hotspots Revealed Potential Candidate Genes Related to Breed Characteristics of Jiangxi Goats

Natural and artificial selection generally result in an increase in ROH frequency (Pemberton et al., 2012), and ROH hotspots are also under positive selection (Xie et al., 2019). In the two Jiangxi goat breeds, genes identified in ROH hotspots were significantly (*p* < 0.05) enriched in several GO terms and KEGG pathways; however, we focused on genes in GO terms and KEGG pathways known to have substantial effects on economically important traits in livestock. Here, ROH hotspots contained many candidate genes associated with breed characteristics, such as fertility, desirable meat quality, and disease resistance in GX and GF goat breeds. In addition, many genes related to skeletal development in ROH hotspots were detected, which may be related to the fact that large body size is one of the current breeding goals for GX and GF goats (Ma et al., 2011), as larger body size increases meat yield and market competitiveness. For GX goats, breed-specific candidate genes were predominantly involved in fertility, growth, fat deposition, and immune response. Genes such as *WNT2*, *EGR1*, *KDM6B*, and *PER1* have been identified as candidate genes for reproduction (Monkley et al., 1996; Tourtellotte et al., 2000; Pilorz and Steinlechner, 2008; Iwamori et al., 2013); the *TIMP3* and *KDM3B* genes have been reported to be associated with growth and skeletal development (Liu et al., 2015; Saw et al., 2019); the loss of genes *FBXO7* and *ACOD1* can lead to impaired immune function (Cordes et al., 2016; Ballesteros Reviriego et al., 2019); and mice lacking *TK2* and *CIDEC* have increased white adipocytes (Nishino et al., 2008; Villarroya et al., 2011). GO terms and KEGG pathways for metabolism, ear development, visual perception and cell differentiation, and stress response were enriched in GF, while gene annotation in ROH regions associated with fertility, hearing and vision, fat deposition, and immune traits supported it. For instance, the genes *ICA1L* and *DENND1A* play important roles in reproductive development (He et al., 2015; Shi et al., 2019); *HES1*, *RELN*, and *SLC26A5* have been reported to be associated with auditory function in humans and mice (Zine and de Ribaupierre, 2002; Wu et al., 2004; Schrauwen et al., 2009); *LRIT3*, *CNGB3*, and *ATXN7* play essential roles in the formation of vision (Yoo et al., 2003; Greenberg et al., 2014; Hasan et al., 2020); *PPP3CA* is related to the intramuscular fat content of pigs (Davoli et al., 2016); mice lacking *ARHGAP5* have a reduced number of adipose cells (Sordella et al., 2003); and *EPS15* is necessary for the immunization of mice (Pozzi et al., 2012).

The overlapping ROH hotspots contain eleven candidate genes between the GX and GF goat breeds, representing the identical genetic characteristics of the two Jiangxi goat breeds. For example, *FAF1* is involved in one of the key signaling pathways required for bone and cartilage formation (Rangkasenee et al., 2013), and deletion of the *FAF1* gene causes early mouse embryonic arrest (Peng et al., 2017). Mice lacking *CHMP5* present polysyndactyly, and failure of tibial and fibular joints is observed (Greenblatt et al., 2015), and *CHMP5* acts as the key regulator of embryonic development (Shim et al., 2006). *CBFB*-knockout mice show severe skeletal developmental defects (Miller et al., 2002). Jiangxi goats are renowned for their high fecundity, and the lambing rates are as high as 300% and 285% in GX and GF goats, respectively (Ma et al., 2011). It was no surprise that common reproduction-related genes were detected, including *B4GALT1* and *BRPF1*, putatively under selection. Mice lacking *B4GALT1* have delayed sperm development and difficulty delivering litters at birth (Lu et al., 1997); *BRPF1* affects the development of embryos and cell division (You et al., 2015). We also identified genes related to immunity that are beneficial for the survival and development of two goat breeds, such as *AQP3*, which is associated with immune loss on mucosal surfaces in knockout mice (Thiagarajah et al., 2017).

Bertolini et al. (2018a) detected the shared ROH region on CHR12 (50–51 Mb) in global goat breeds (no Chinese) using the sliding window method in PLINK software (Chang et al., 2015). In this study, we confirmed and complemented a family of sensory organ genes (*GJA3*, *GJB2*, and *GJB6*) associated with gap junctions in an ROH hotspot on CHR12 (50.55–50.81 Mb) in Chinese and other goat breeds worldwide (including the wild ancestor, the bezoar) *via* the consecutive method in the R package *detectRUNS* (Biscarini et al., 2019). *GJA3* variants are related to cataracts in humans (Berthoud et al., 2014). *GJB2* and *GJB6* play crucial roles in hearing (Teubner, 2003; Prera et al., 2014). This functional region is located in a common ROH hotspot among Chinese sheep breeds (Abied et al., 2020), and these three genes are identical candidate genes under positive selection in Boer goats (Onzima et al., 2018), Barki goats, and sheep (Kim et al., 2016). Goat domestication occurred in Fertile Crescent around 12,000–10 ,000 years BP, which involved the bezoar (*Capra aegagrus*) as the wild ancestor (Zeder, 2008). This perception-related genome region might have been selected before domestication in goats and under parallel selection between goats and sheep because they need better senses of sight and hearing to feed and detect the presence of enemies in the wild. GF goats have more candidate genes associated with hearing and vision mentioned above, possibly because GF goats are easily discovered by their white coats. In this case, GF goats need to possess more sensitive sight and hearing to avoid potential dangers. These findings provide some theoretical basis for future research on the molecular mechanisms underlying the germplasm characteristics of Jiangxi goats.

### ROHet Hotspots Uncovered Candidate Genes Possibly Under Balancing Selection

In livestock, ROHet hotspots are far less characterized than ROH hotspots (Biscarini et al., 2020). Williams et al. (2016) identified that although about 90% of the genome was covered by ROH in Chillingham cattle, some regions were highly heterozygous. The lower proportion of ROHet indicated that they may not be important participants in adaptation. However, heterozygous-enriched regions can prevent the accumulation of deleterious homozygotes, which may have a positive impact on adaptation and traits (Santos et al., 2021). In addition, a growing body of research has suggested that the role of ROHet hotspot regions on biodiversity and adaptive function cannot be overlooked (Williams et al., 2016; Santos et al., 2021). In two Jiangxi goat breeds, growth-related (*NBEA* and *OSR2*) and fertility-related (*STAG1*, *PCCB*, and *CSMD1*) genes were explored. *NBEA-*knockout mice present growth deficiency (Nuytens et al., 2014); the *OSR2* gene is related to palatal bone formation (Fu et al., 2017), and a lack of *OSR2* results in early tooth developmental arrest in mice (Zhou et al., 2011); and *CSMD1*-knockout mice show increased rates of infertility (Lee et al., 2019). We highlighted that global domesticated goat breeds (except for bezoars) shared an ROHet hotspot (CHR1:132.21–132.54 Mb) under balancing selection, as validated by Tajima’s *D* and BetaScan2 statistics. Two reproduction-related genes, *STAG1* and *PCCB*, were found in this region. Mice homozygous for a gene-trap allele of *STAG1* exhibit embryonic lethality (Remeseiro et al., 2012). *PCCB* plays important roles in embryonic development (Ortega et al., 2017). This genome area might be caused by long-term balancing selection after domestication and may be indispensable for domesticated goats.

## Conclusion

In conclusion, this work investigated the genome-wide ROH, ROHet, and genetic load of two indigenous goat breeds from Jiangxi Province, China, in a global panel of goats. The results showed that GF goats had more abundant genomic heterozygosity than GX goats; however, GF goats harbored a higher fraction of damaging homozygotes in ROHs than that of GX goats. The putative damaging variants were more enriched in long ROHs in the goat genome. A series of genes associated with growth, fertility, or/and environmental adaptation traits were detected in the ROH and ROHet hotspots of GF and GX goats. In addition, we highlighted that a sense-related ROH hotspot and reproduction-related ROHet hotspot were shared among goat breeds worldwide and may play important roles in the adaptive evolution of goats.

## Data Availability

The datasets presented in this study can be found in online repositories. The names of the repository and accession number can be found at: https://doi.org/10.6084/m9.figshare.14748180.v1.
