# Supplementary figures and images for: Genome-Wide Estimates of Runs of Homozygosity, Heterozygosity, and Genetic Load in Two Chinese Indigenous Goat Breeds
Source: Front Genet. 2022 Apr 26;13:774196. doi: 10.3389/fgene.2022.774196 (PMC9086400; doi:10.3389/fgene.2022.774196)

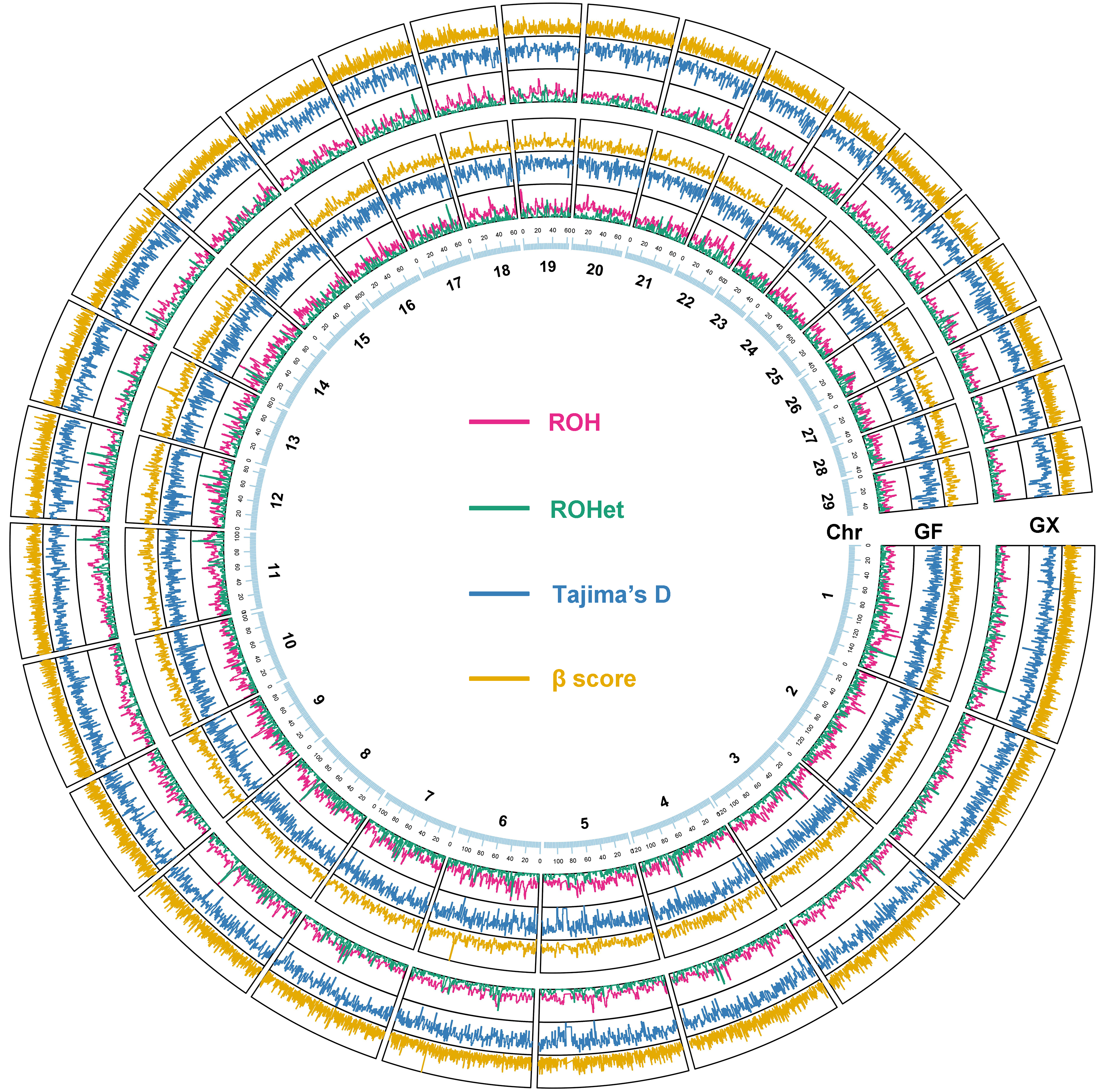

Supplement: Supplementary file 1 [file Image3.TIF]

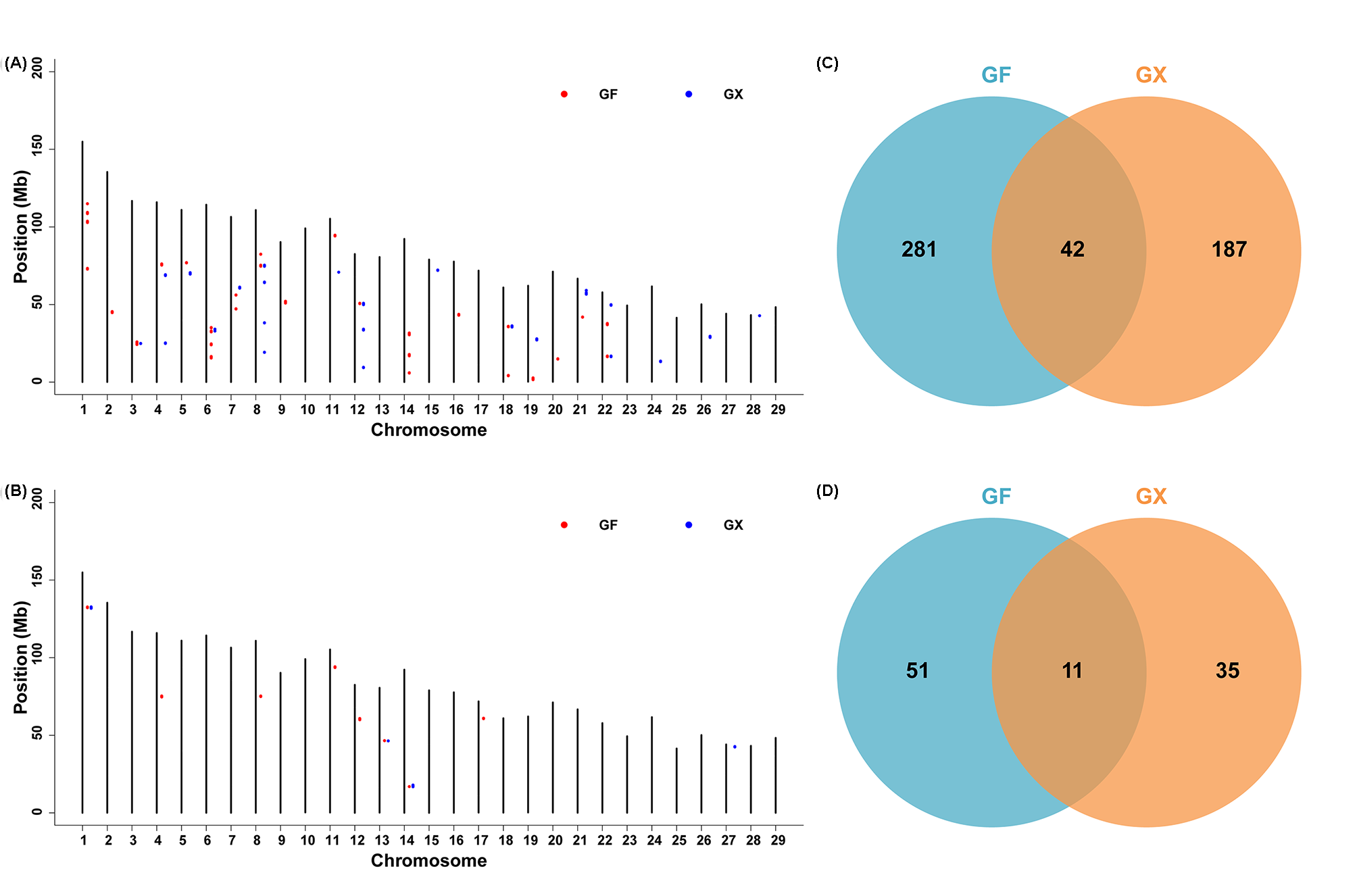

Supplement: Supplementary file 2 [file Image2.TIF]

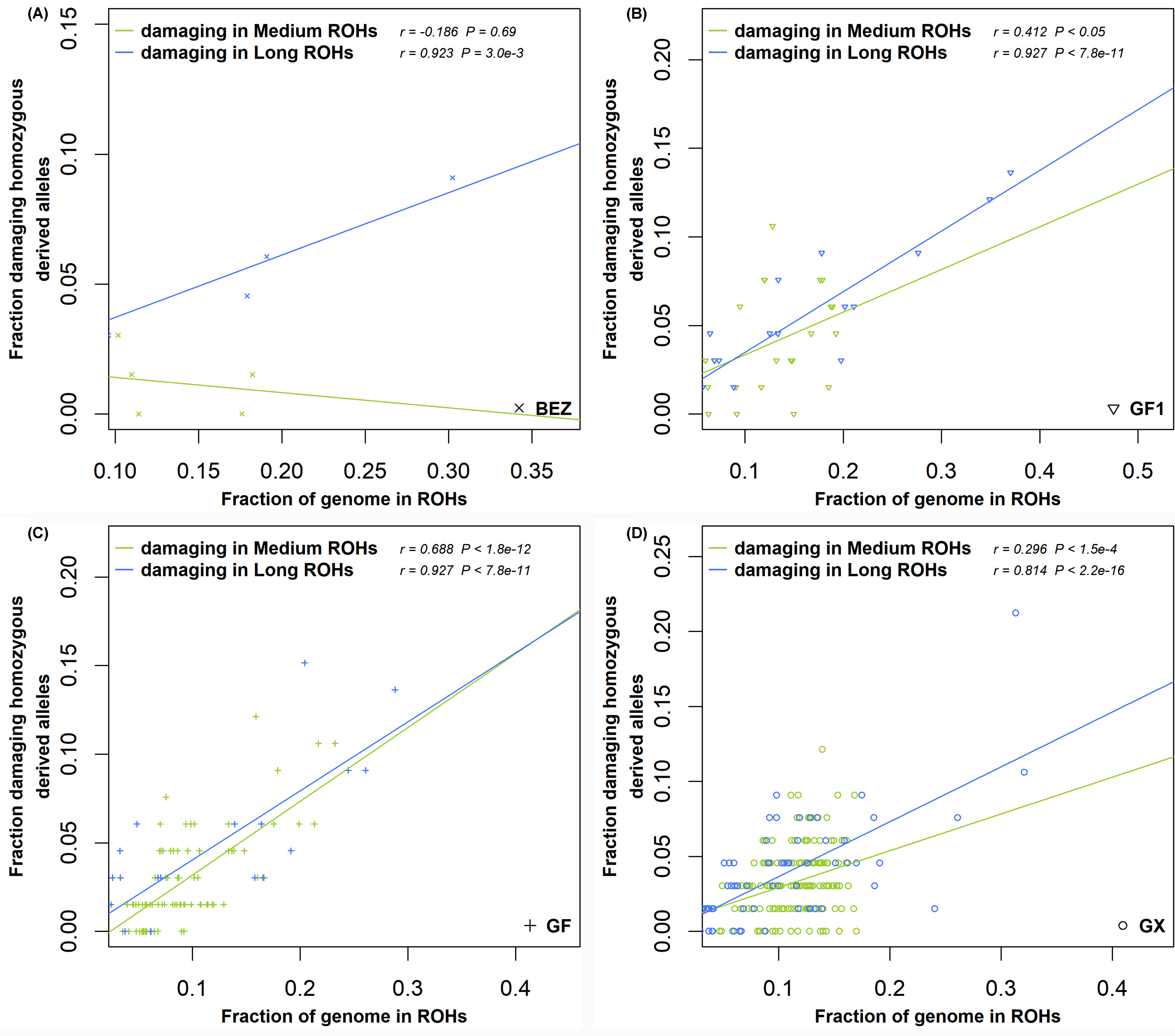

Supplement: Supplementary file 3 [file Image1.TIF]
